# Paralemnolide A, an Unprecedented Bisnorsesquiterpene from the Taiwanese Soft Coral *Paralemnalia thyrsoides*

**DOI:** 10.3390/md10071528

**Published:** 2012-07-17

**Authors:** Shang-Kwei Wang, Yu-Sheng Lee, Chang-Yih Duh

**Affiliations:** 1 Asia-Pacific Ocean Research Center, National Sun Yat-Sen University, Kaohsiung 804, Taiwan; Email: skwang@cc.kmu.edu.tw; 2 Department of Microbiology, Kaohsiung Medical University, Kaohsiung 807, Taiwan; 3 Department of Marine Biotechnology and Resources, National Sun Yat-Sen University, Kaohsiung 804, Taiwan; Email: m995020028@student.nsysu.edu.tw

**Keywords:** *Paralemnalia thyrsoides*, paralemnolide A, cytotoxicity, anti-HCMV

## Abstract

Paralemnolide A (**1**), possessing an unprecedented bisnorsesquiterpene skeleton, was isolated from the soft coral *Paralemnalia thyrsoides*. The structure of paralemnolide A was elucidated by extensive analysis of spectroscopic data. The anti-HCMV (human cytomegalovirus) activity of **1** and its cytotoxicity against selected cell lines were evaluated.

## 1. Introduction

**Figure 1:**
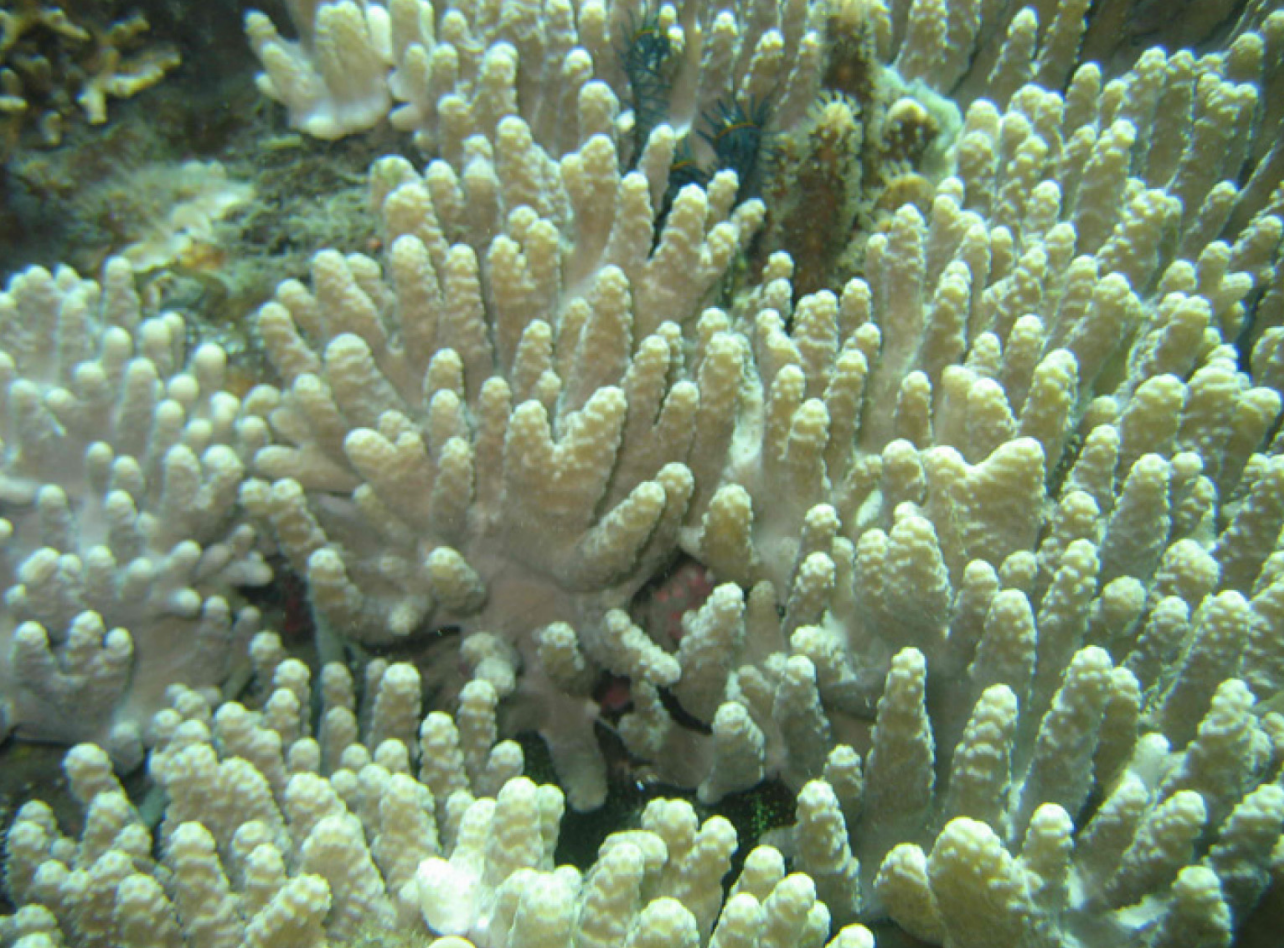
Soft coral *Paralemnalia thyrsoides*.

**Figure 2:**
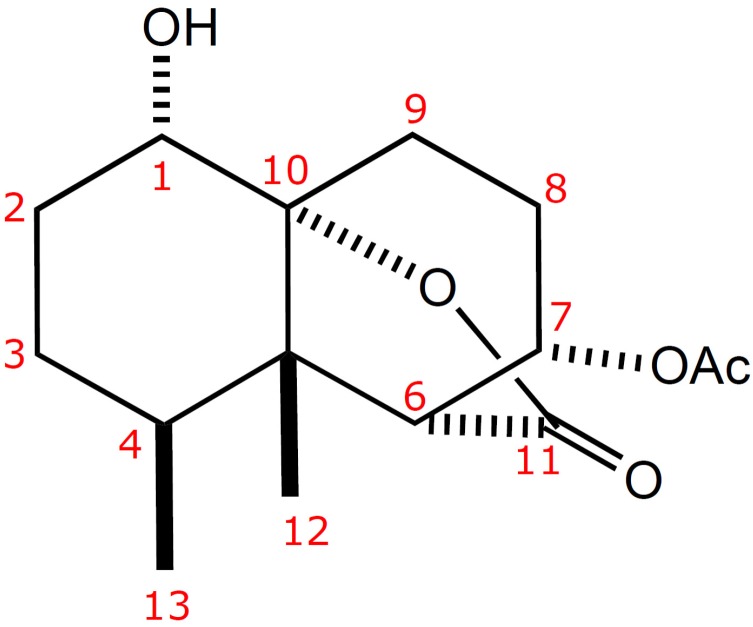
Structure of Paralemnolide A (**1**).

Soft corals of the genus *Paralemnalia* [1,2,3,4,5,6,7,8] have been found to be rich sources of sesquiterpenoids of nardosinane [1,2,3], neolemnane [3,4] and africanane-type [3] compounds, as well as norsesquiterpenoids of nornardosinane-type [3,5,6] compounds. As part of the continuing search for bioactive substances from marine invertebrates, we explored the secondary metabolites of the soft coral *Paralemnalia thyrsoides* (Ehrenberg, 1934) which was collected from Sansiantai, Taitong County, Taiwan (Figure 1). Chromatographic separation on the acetone extract of the soft coral *P. thyrsoides* resulted in the isolation of paralemnolide A (**1**), which possesses an unprecedented bisnorsesquiterpene skeleton (Figure 2). The anti-HCMV (human cytomegalovirus) activity of **1** and its cytotoxicity against P-388 (mouse lymphocytic leukemia), HT-29 (human colon adenocarcinoma), and A549 (human lung carcinoma) cancer cell lines were evaluated *in vitro*.

## 2. Results and Discussion

The acetone extract of *P. thyrsoides* was concentrated to a brown gum, which was partitioned between EtOAc and H_2_O. The EtOAc-soluble residue (20 g) was subjected to a series of chromatographic separations to yield **1**.

Paralemnolide A (**1**) was obtained as a colorless oil. The HRESIMS of **1** exhibited a [M + Na]^+^ peak at *m/z* 305.1367, consistent with the molecular formula of C_15_H_22_O_5_, implying five degrees of unsaturation. The ^13^C NMR and DEPT spectra showed resonances for three methyls, four methylenes, four methines, and four quaternary carbons. The absorptions at 3447, 1769, 1742 cm^−1^ in its IR spectrum revealed the presence of hydroxyl and carbonyl groups. The ^1^H and ^13^C NMR spectra (Table 1) showed the presence of the following groups: (a) a secondary methyl (*δ*_C_ 15.8 CH_3_; *δ*_H_ 0.85 d, *J =* 6.8 Hz), (b) a tertiary methyl (*δ*_C_ 10.4 CH_3_; *δ*_H_ 1.03 s), (c) two oxygenated methines (*δ*_C_ 69.8 CH; *δ*_H_ 3.46 dd, *J =* 11.6, 5.2 Hz and *δ*_C_ 66.7 CH; *δ*_H_ 5.17 ddd, *J =* 10.8, 6.8, 2.4 Hz), and (d) an acetyl group (*δ*_C_ 170.6 C, 21.2 CH_3_; *δ*_H_ 2.09 s).

**Table 1:** ^1^H and ^13^C NMR Spectroscopic Data of **1**^a^.

C/H	^13^C		^1^H
1	69.8	CH ^b^	3.46 dd (11.6, 5.2) ^c^
2α	30.5	CH_2_	1.63 m
2β			1.95 m
3α	27.8	CH_2_	1.59 m
3β			1.35 m
4	35.0	CH	1.59 m
5	47.9	qC	
6	53.3	CH	2.70 d (2.4)
7	66.7	CH	5.17 ddd (10.8, 6.8, 2.4)
8α	24.2	CH_2_	1.63 m
8β			2.24 m
9α	25.3	CH_2_	2.57 m
9β			1.73 m
10	89.2	qC	
11	175.0	qC	
12	10.4	CH_3_	1.03 s
13	15.8	CH_3_	0.85 d (6.8)
OAc	170.6	qC	
	21.2	CH_3_	2.09 s

^a^ Spectra were measured in CDCl_3_ (^1^H, 400 MHz and ^13^C, 100 MHz); ^b^ Multiplicities are deduced by HSQC and DEPT experiments; ^c^*J* values (in Hz) are in parentheses.

**Figure 3:**
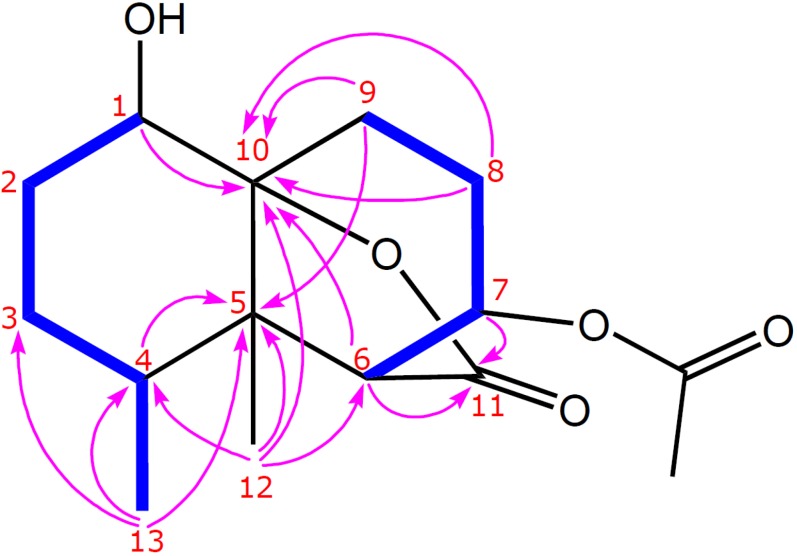
Key ^1^H–^1^H COSY (▬) and HMBC (H→C) correlations of **1**.

By interpretation of ^1^H–^1^H COSY correlations (Figure 3), it was possible to establish two partial structures of consecutive proton systems extending from H-1 to Me-13 through H-2, H-3, and H-4 as well as from H-6 to H_2_-9 through H-7 and H-8. HMBC correlations of (a) C*H*_3_-13 to C-3, C-4, and C-5, (b) C*H*_3_-12 to C-4, C-5, C-6, and C-10, (c) H-6 and H-7 to the carbonyl C-11 atom, and (d) H-1, H-6, and H-9 to C-10 connected the latter two spin systems concluding the planar structure of **1**, as shown in Figure 3. The above functionalities revealed that paralemnolide A (**1**) possesses a novel bisnorsesquiterpene tricyclic skeleton. 

**Figure 4:**
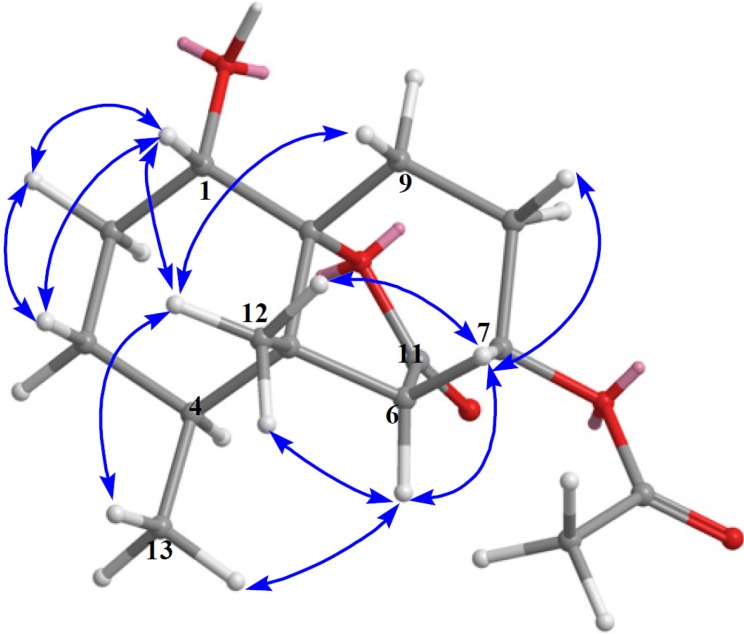
Selected NOESY correlations of **1**.

**Figure 5:**
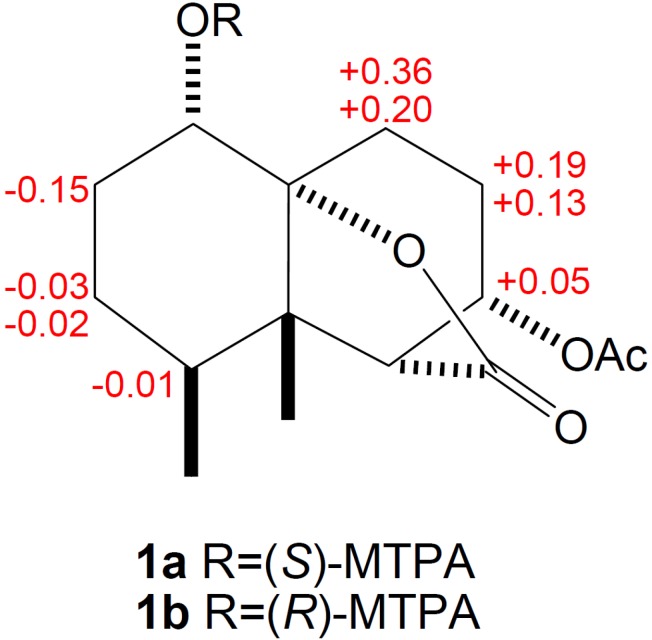
Absolute stereochemistry of **1**: values (δ*_S_* − δ*_R_*) in ppm for the two MTPA esters **1a** and **1b**.

The relative configuration of **1** was deduced from a NOESY experiment. NOE correlations of H-1/H_3_-12, H-6/H_3_-12, H-6/H_3_-13, and H-7/H_3_-12 suggested all to be on the β-side of the molecule (Figure 4).The absolute configuration of **1** was determined by application of the modified Mosher method [9]. Treatment of **1** with (*R*)-MTPA chloride and (*S*)-MTPA chloride afforded the (*S*)-MTPA ester (**1a**) and (*R*)-MTPA ester (**1b**), respectively. The difference in chemical shift values (δ*_S_* − δ*_R_*) for the diastereomeric esters **1a** and **1b** was calculated in order to assign the absolute configuration at C-1. Calculations for all of the relevant signals suggested the 1*S* absolute configuration. Therefore, the 4*S*, 5*S*, 6*R*, 7*S*, and 10*R* absolute configuration was proposed for **1** on the basis of the ∆δ results summarized in Figure 5.

**Scheme 1:**
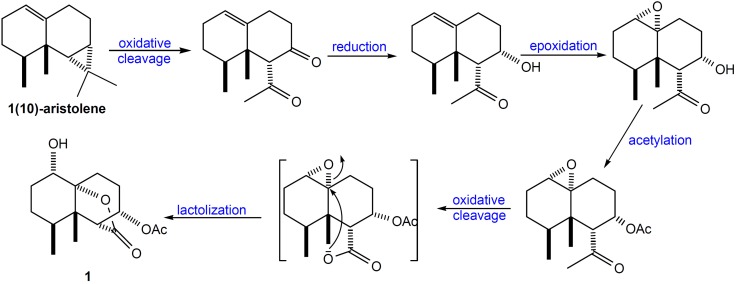
Possible Biogenetic Pathway for **1**.

It is worthwhile to mention that the framework of **1** may be derived from 1(10)-aristolene through a sequence of oxidative cleavage, reduction, epoxidation, oxidative cleavage, and lactonization to result in the formation of paralemnolide A (**1**) as depicted in Scheme 1.

Paralemnolide A (**1**) was evaluated for cytotoxicity against P-388, A549, and HT-29 cancer cell lines. Metabolite **1** displayed moderate cytotoxicity against P-388, with an ED_50_ of 3.8 μg/mL. With the exception of the above finding, the obtained negative result showed that **1** was not cytotoxic against A549, and HT-29 cancer cell lines (ED_50_ > 50 μg/mL). The compound was also examined for antiviral activity against human cytomegalovirus (HCMV) using a human embryonic lung (HEL) cell line. Paralemnolide A (**1**) did not show anti-HCMV activity.

## 3. Experimental Section

### 3.1. General Experimental Procedures

Optical rotations were determined with a JASCO P1020 digital polarimeter. UV and IR spectra were obtained on JASCO V-650 and JASCO FT/IR-4100 spectrophotometers, respectively. NMR spectra were recorded on a Varian MR 400 NMR spectrometer at 400 MHz for ^1^H and 100 MHz for ^13^C, respectively. ^1^H NMR chemical shifts are expressed in *δ* (ppm) referring to the solvent peak *δ*_H_ 7.27 for CDCl_3_, and coupling constants are expressed in Hz. ^13^C NMR chemical shifts are expressed in *δ* (ppm) referring to the solvent peak *δ*_C_ 77.0 for CDCl_3_. MS were recorded by a Bruker APEX II mass spectrometer. Silica gel 60 (Merck, Germany, 230–400 mesh) and LiChroprep RP-18 (Merck, 40–63 μm) were used for column chromatography. Precoated silica gel plates (Merck, Kieselgel 60 F_254_, 0.25 mm) and precoated RP-18 F_254s_ plates (Merck) were used for thin-layer chromatography (TLC) analysis. High-performance liquid chromatography (HPLC) was carried out using a Hitachi L-7100 pump equipped with a Hitachi L-7400 UV detector at 220 nm together with a semi-preparative reversed-phase column (Merck, Hibar LiChrospher RP-18e, 5 μm, 250 × 25 mm).

### 3.2. Biological Material

The octocoral *P. thyrsoides* was collected by hand using scuba at the Sansiantai, Taitong County, Taiwan, in July 2008, at a depth of 6 m. This soft coral was identified by Prof. Chang-Fong Dai, Institute of Oceanography, National Taiwan University. A voucher specimen (SST-07) was deposited in the Department of Marine Biotechnology and Resources, National Sun Yat-sen University.

### 3.3. Extraction and Isolation

The frozen soft coral was chopped into small pieces and extracted with acetone in a percolator at room temperature. The acetone extract of *P. thyrsoides* was concentrated to a brown gum, which was partitioned with EtOAc and H_2_O. The EtOAc-soluble residue (20 g) was subjected to Si 60 CC using *n*-hexane–EtOAc mixtures of increasing polarity for elution. Fractions eluted by *n*-hexane–EtOAc (2:1) were further purified by RP-18 HPLC [eluted with MeOH–H_2_O (1:1)] to yield **1** (2.2 mg).

Paralemnolide A (**1**): colorless, viscous oil; [α]_D_^25^ −25 (*c* 0.4, CHCl_3_); IR (neat) ν_max_ 3447, 3038, 2964, 1769, 1456, 1379, 1239 cm^−1^; ^1^H NMR and ^13^C NMR data, see Table 1; HRESIMS *m/z* 305.1367 [M + Na]^+^ (calcd. for C_15_H_2__2_O_5_Na, 305.1365).

Preparation of Mosher’s esters of **1**. In separate NMR tubes, duplicate (0.7 mg) samples of **1** were dissolved in 0.6 mL of pyridine-*d*_5_ and allowed to react for 3 h at room temperature with (*R*)- and (*S*)-MTPA chloride (one drop) to yield (*S*)-MTPA ester **1a** and (*R*)-MTPA ester **1b**, respectively. ^1^H-NMR (pyridine-*d*_5_, 400 MHz) of **1a**: *δ*_H_ 0.67 (3H, d, *J* = 6.8 Hz, Me-13), 0.99 (3H, s, Me-12), 1.32 (1H, m, H-3α), 1.35 (1H, m, H-3β), 1.57 (1H, m, H-4), 1.74 (1H, m, H-8α), 1.76 (1H, m, H-2α), 1.91 (1H, m, H-2β), 1.96 (1H, m, H-9β), 2.27 (1H, m, H-8β), 2.28 (1H, m, H-9α), 3.00 (1H, d, *J* = 2.4 Hz, H-6), 5.27 (1H, dd, *J* = 11.6, 5.2 Hz, H-1), 5.31 (1H, ddd, *J* = 10.8, 7.6, 3.2 Hz, H-7), 7.46–7.66 (5H, m, Ph). ^1^H-NMR (pyridine-*d*_5_, 400 MHz) of **1b**: *δ*_H_ 0.67 (3H, d, *J* = 7.2 Hz, Me-13), 0.97 (3H, s, Me-12), 1.35 (1H, m, H-3α), 1.37 (1H, m, H-3β), 1.55 (1H, m, H-8α), 1.58 (1H, m, H-4), 1.76 (1H, m, H-9β), 1.86 (H, m, H-2α), 1.92 (1H, m, H-9α), 2.03 (H, m, H-2β), 2.14 (1H, m, H-8β), 2.97 (1H, d, *J* = 2.0 Hz, H-6), 5.23 (1H, dd, *J* = 11.6, 4.80 Hz, H-1), 5.26 (1H, ddd, *J* = 10.8, 6.4, 3.2 Hz, H-7), 7.45–7.65 (5H, m, Ph).

### 3.4. Cytotoxicity Assay

Cytotoxicity was determined on P-388 (mouse lymphocytic leukemia), HT-29 (human colon adenocarcinoma), and A-549 (human lung epithelial carcinoma) tumor cells using a modification of the MTT colorimetric method according to a previously described procedure [10,11]. The provision of the P-388 cell line was supported by J.M. Pezzuto, formerly of the Department of Medicinal Chemistry and Pharmacognosy, University of Illinois at Chicago. HT-29 and A-549 cell lines were purchased from the American Type Culture Collection. To measure the cytotoxic activities of tested compounds, five concentrations with three replications were performed on each cell line. Mithramycin was used as a positive control.

### 3.5. Anti-HCMV Assay

To determine the effects of natural products upon HCMV cytopathic effect (CPE), confluent human embryonic lung (HEL) cells grown in 24-well plates were incubated for 1 h in the presence or absence of various concentrations of tested natural products with three replications. Ganciclovir was used as a positive control. Then, cells were infected with HCMV at an input of 1000 pfu (plaque forming units) per well of a 24-well dish. Antiviral activity was expressed as IC_50_ (50% inhibitory concentration), or compound concentration required to reduce virus induced CPE by 50% after 7 days as compared with the untreated control. To monitor the cell growth upon treating with natural products, an MTT-colorimetric assay was employed [12].

## 4. Conclusion

The first investigation of soft coral *P. thyrsoides* collected at San-Hsian-Tai (Taitong County, Taiwan) has led to the isolation of an unprecedented bisnorsesquiterpene, paralemnolide A (**1**) exhibiting cytotoxicity against P-388 cell line with ED_50_ of 3.8 μg/mL. 

## Supplementary Files

Supplementary File 1: PDF-Document (PDF, 597 KB)
