# Supplementary material for: Paralemnolide A, an Unprecedented Bisnorsesquiterpene from the Taiwanese Soft Coral Paralemnalia thyrsoides
Source: Mar Drugs. 2012 Jul 17;10(7):1528–35. doi: 10.3390/md10071528 (PMC3407928; doi:10.3390/md10071528)

Supplementary Materials

Table of Contents

Figure S1. ^{1}H NMR spectrum (400 MHz) of paralemnolide A (**1**) in $CDCl_3$. 2

Figure S2. ^{13}C NMR spectrum (100 MHz) of paralemnolide A (**1**) in $CDCl_3$. 3

Figure S3. COSY spectrum (400 MHz) of paralemnolide A (**1**) in $CDCl_3$. 4

Figure S4. HSQC spectrum (400 MHz) of paralemnolide A (**1**) in $CDCl_3$ 5

Figure S5. HMBC spectrum (400 MHz) of paralemnolide A (**1**) in $CDCl_3$. 6

Figure S6. NOESY spectrum (400 MHz) of paralemnolideA (**1**) in $CDCl_3$. 7

Figure S1. ^{1}H NMR spectrum (400 MHz) of paralemnolide A (**1**) in $CDCl_3$.

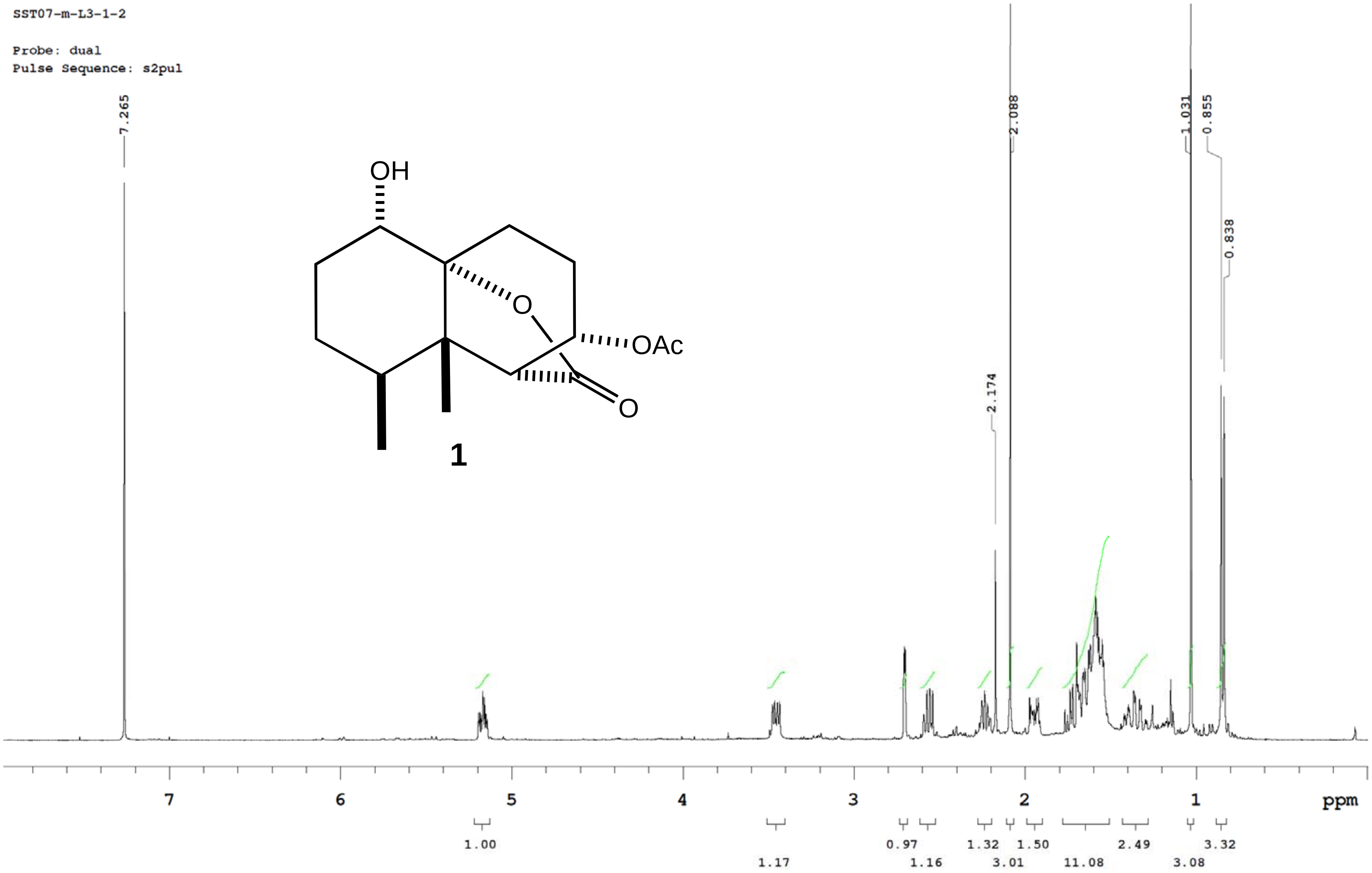

Figure S2. ^{13}C NMR spectrum (100 MHz) of paralemnolide A (**1**) in $CDCl_3$.

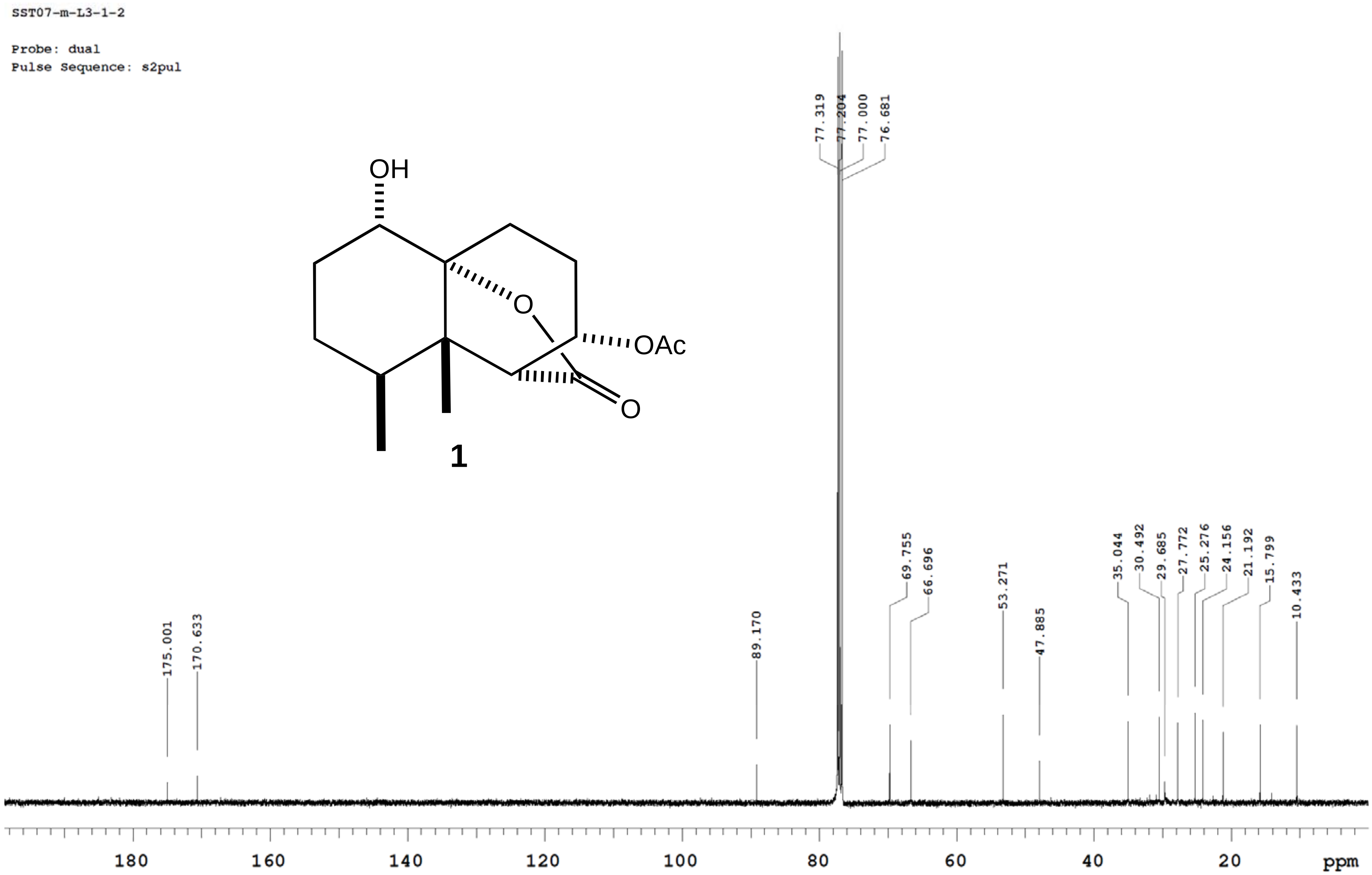

Figure S3. $^1H-^1H$ COSY spectrum (400 MHz) of paralemnolide A (**1**) in $CDCl_3$.

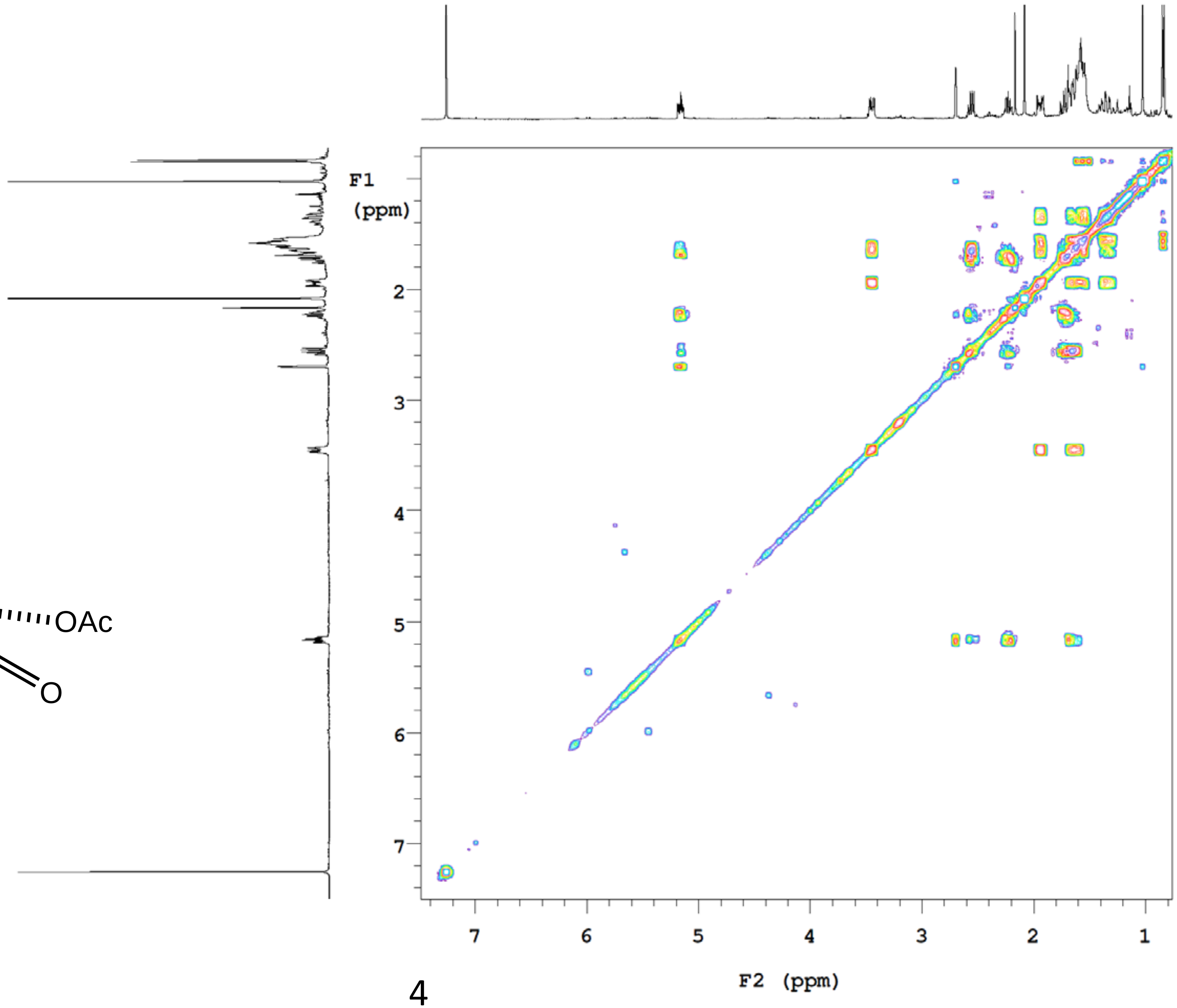

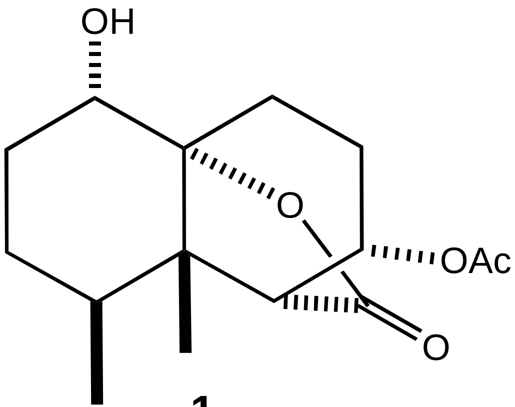

1

Figure S4. HSQC spectrum (400 MHz) of paralemnolide A (**1**) in $CDCl_3$.

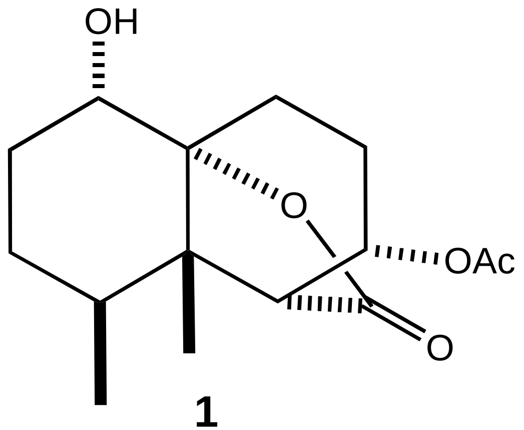

1

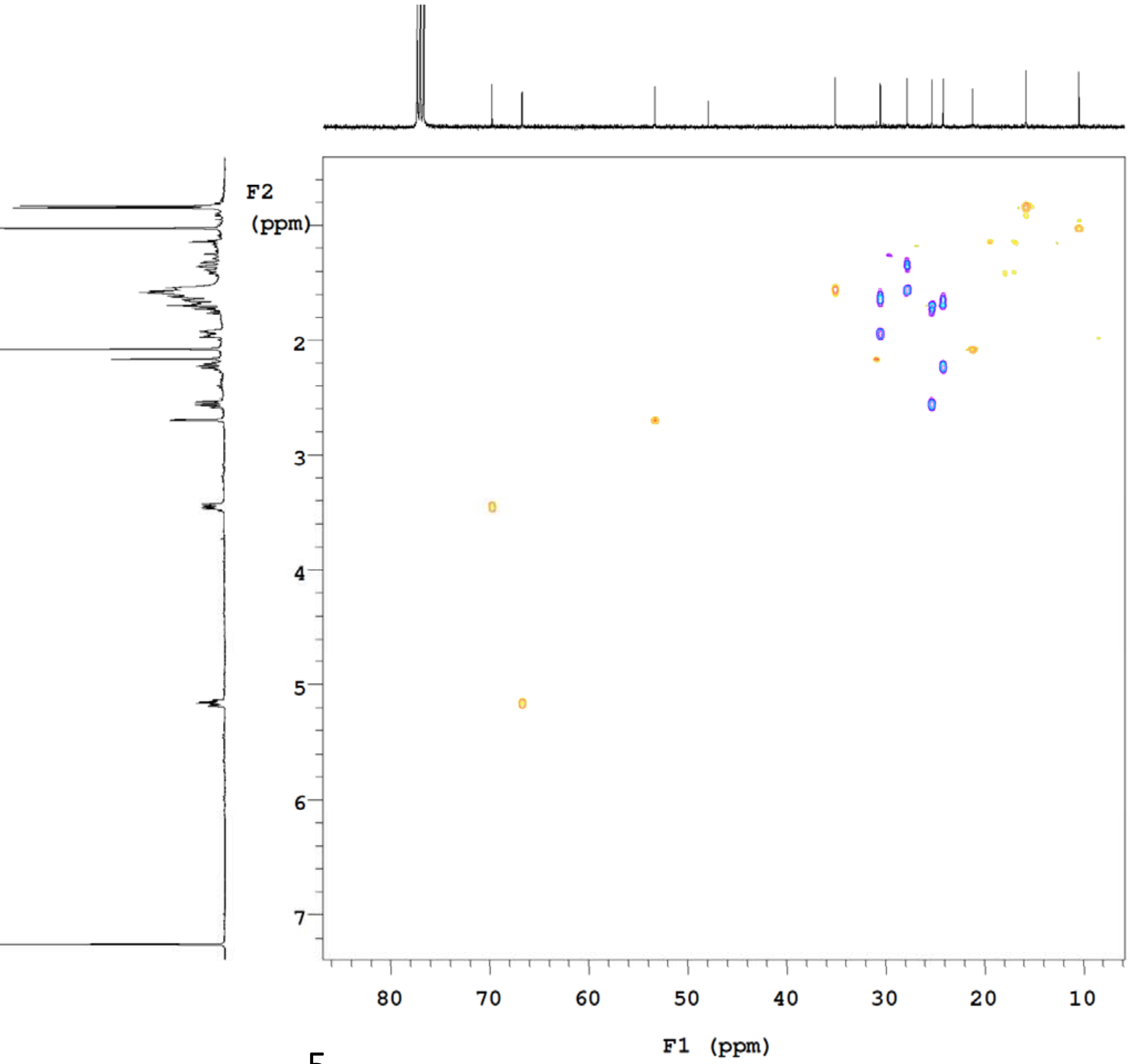

Figure S5. HMBC spectrum (400MHz) of paralemnolide A (**1**) in $CDCl_3$.

SST07-m-L3-1-2

Probe: dual
Pulse Sequence: gHMBC

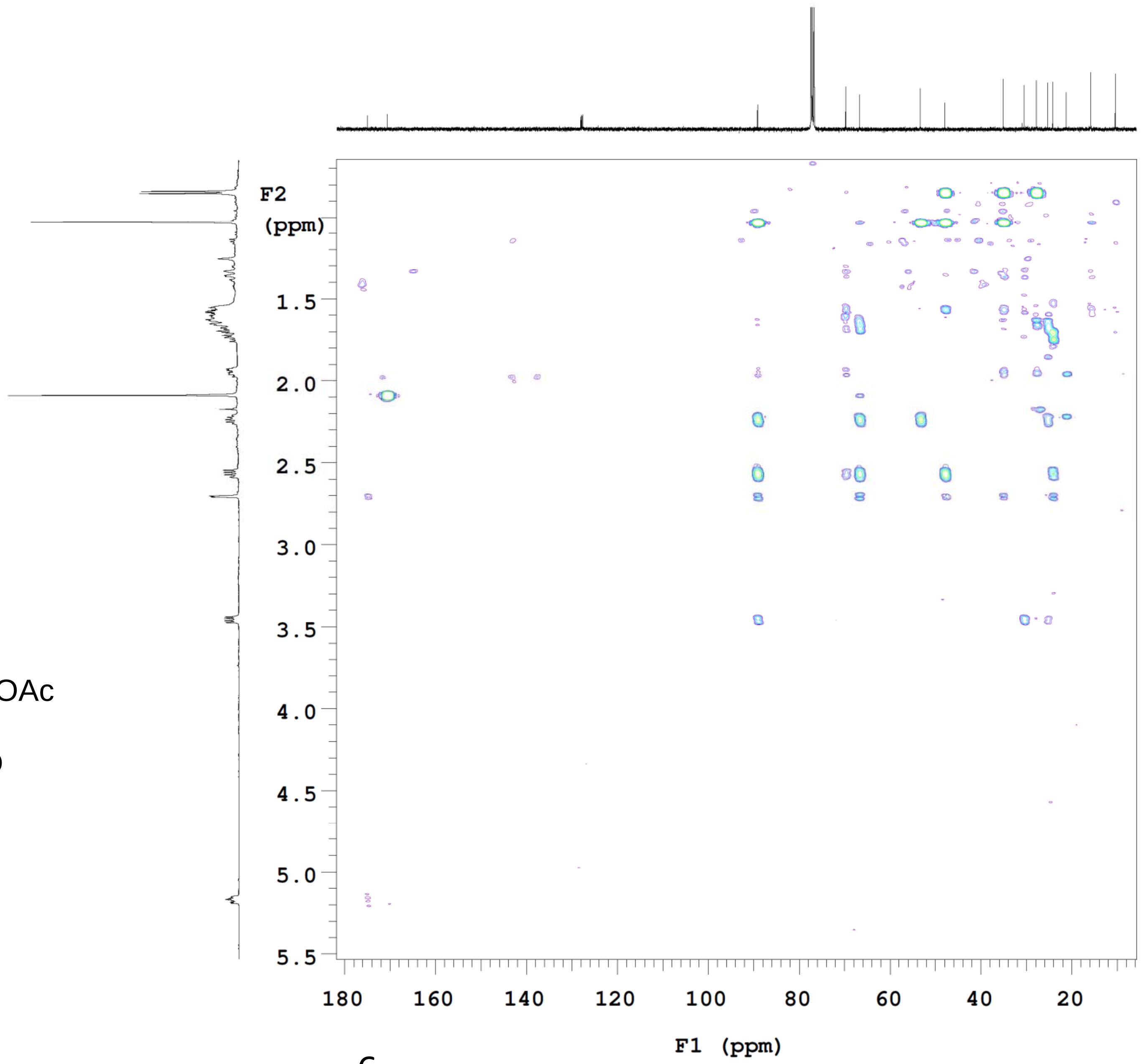

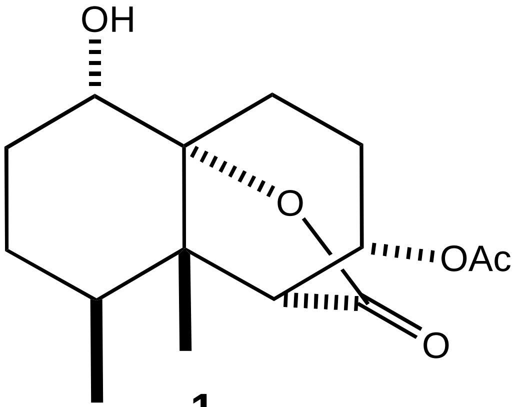

1

Figure S6. NOESY spectrum (400 MHz) of paralemnolide A (**1**) in $CDCl_3$.

SST07-m-L3-1-2

Probe: dual
Pulse Sequence: NOESY

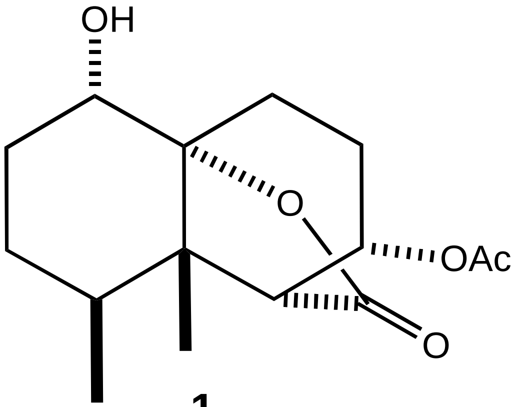

1

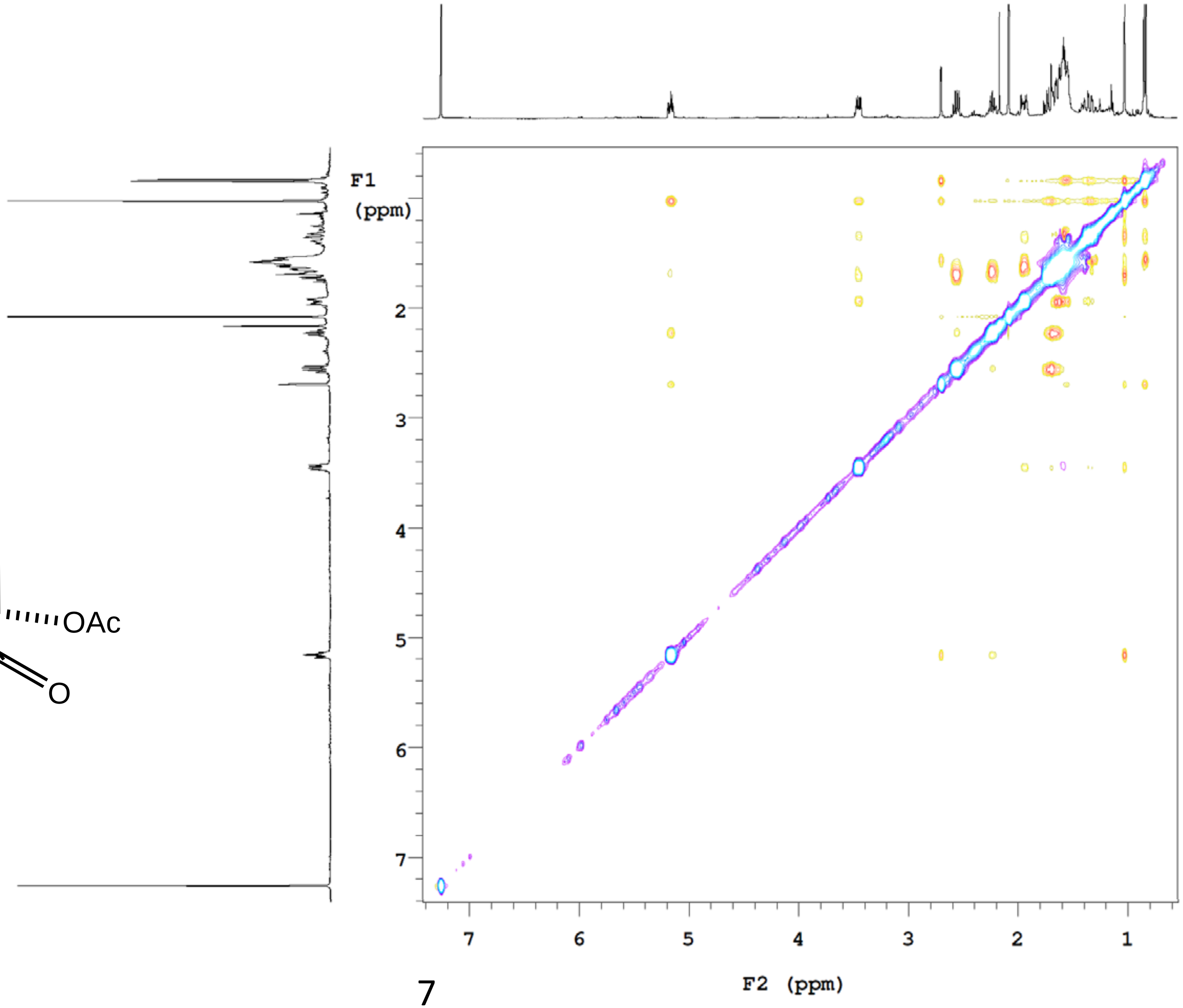

Supplement: Supplementary File 1: — PDF-Document (PDF, 597 KB) [file marinedrugs-10-01528-s001.pdf]
